# Long-term safety assessment in children who received hydrolyzed protein formulas with *Lactobacillus rhamnosus* GG: a 5-year follow-up

**DOI:** 10.1007/s00431-016-2825-4

**Published:** 2016-12-15

**Authors:** DMF Scalabrin, C Harris, WH Johnston, CL Berseth

**Affiliations:** 1 Clinical Research, Department of Medical Affairs, Mead Johnson Nutrition, Evansville, IN 47712 USA; 2 Birmingham Pediatric Group, Birmingham, AL USA

**Keywords:** LGG, Probiotics, Hydrolyzed formula, Allergic manifestations, Children

## Abstract

Extensively hydrolyzed (EH) formula with *Lactobacillus rhamnosus* GG (LGG) was demonstrated to alleviate cow’s milk allergy (CMA) symptoms and promote faster acquisition of tolerance to cow’s milk protein. We previously demonstrated that partially hydrolyzed (PH) and EH formulas with LGG supported normal growth in healthy-term infants through 120 days of age. The objective of the current study was to evaluate growth, development, and specific adverse events through 5 years of age in participants from that cohort who continued receiving study formula. Infants who completed a double-blind, randomized growth and tolerance study were eligible to continue receiving the assigned study formula through 1 year of age (control: EH casein formula, EHF, or one of two investigational formulas: EH casein formula with LGG (EHF-LGG) or a PH formula with LGG (PHF-LGG)) and participate in follow-up through 5 years of age. Anthropometric measures, behavior development, and specific adverse events were recorded. No significant differences in achieved weight and height or behavioral development outcomes at 3 or 5 years of age were observed among study groups. Few statistically significant differences in the incidence of specific infection-related events through years 3 or 5 were observed among study groups, none of which were considered clinically relevant.

*Conclusion*: Extensively and partially hydrolyzed formulas with LGG were associated with normal growth and development and long-term safety through 5 years of age.

**Table:** 

**What is Known:** • *Infants with cow’s milk allergy often experience allergic manifestations that can lead to poor nutrition status and poor growth*. *• Providing partially hydrolyzed* (*PH*) *and EH formulas with or without LGG in infants can support normal growth in healthy-term infants*.
**What is New:** *• This study provides long-term safety data for the first 5 years of life on the use of extensively and partially hydrolyzed formulas with LGG when fed through 1 year of age*. *• Extensively and partially hydrolyzed formulas with LGG are associated with normal growth*, *development*, *and long-term safety through 5 years of age*.

## Introduction

Cow’s milk allergy (CMA) affects ∼3% of infants [1, 2] who have immediate (IgE-mediated) or delayed onset (non-IgE-mediated) reactions to cow’s milk protein (CMP), commonly manifested as atopic dermatitis (AD), urticaria, wheezing/asthma, allergic rhinitis, or diarrhea [1, 3]. The majority of infants and children with CMA have two or more symptoms that significantly impact quality of life [4]. Although 80% of children affected can tolerate intact CMP by 3 years of age, it may take up to 5 years or longer for those with IgE-mediated reactions to achieve tolerance [3]. In addition, these children are at higher risk of subsequent development of allergy to other foods and/or inhalants [5–7]. The current management of CMA is based on complete avoidance of intact CMP [1, 3]. Extensively hydrolyzed (EH) cow’s milk-based formulas with demonstrated reduced allergenicity are recommended for infants with CMA [8–10]. Efficacy to manage infants and children with CMA has been demonstrated for EH casein formula [11–13]. There are many risk factors for poor growth in children with CMA, including the impact of the allergic manifestations on nutritional status, gut inflammation causing impaired nutrient absorption, and restricted diet [14]. For example, more children with CMA consume dietary calcium below the recommendations compared with children without CMA [15]. Moreover, use of a hydrolyzed whey formula was associated with slower weight and length growth in infants with CMA (compared to an amino acid-based formula) [16]. Therefore, assessment of growth and development with EH formulas is a critical safety parameter, especially when a probiotic is added to the formula, since there is limited data on growth with probiotic-supplemented formulas [17].

Probiotics are live microorganisms that, when administered in adequate amounts, have been shown in properly controlled studies to confer a health benefit on the host [18]. Mechanisms of action include transitory colonization of the intestinal tract [19, 20], increase of anti-inflammatory cytokines, and stabilization of tight junctions in the gut [21]. Recent data suggest that *Lactobacillus rhamnosus* GG (LGG), the most studied probiotic, may not only impact the host intestinal mucosa directly through adhesion and immunomodulatory interactions [22] but also stimulate anti-inflammatory pathways and butyrate production in the gut resident bacteria [19, 23]. Recent meta-analyses concluded that probiotics, notably *Lactobacillus*, reduce risk of antibiotic-associated diarrhea [24] and may decrease incidence of acute respiratory infections [25, 26]. Furthermore, current ESPGHAN guidelines recommend the use of LGG in children at risk of antibiotic-associated diarrhea [27]. Specific probiotics, especially LGG, may also play a role in preventing or managing atopic eczema [28, 29]. Infants with CMA who received EH formula with LGG demonstrated improved gut barrier function and experienced a faster acquisition of tolerance to CMP [23, 28, 30–32]. Although safety issues associated with the use of live probiotic bacteria are rare and have been reported only in immunocompromised or seriously ill individuals, there are still safety concerns associated with the use of live probiotic bacteria [33, 34].

Consequently, continued evaluation of hydrolyzed formulas with LGG for infant growth and development, as well as long-term safety after early-life usage, is warranted. We previously demonstrated that partially hydrolyzed (PH) and EH formulas with or without LGG support normal growth in a cohort of healthy-term infants with no differences in formula tolerance and immune markers through 120 days of age [35]. In the current study, we evaluated growth and development, specific allergic and infectious events, and serious adverse events through 5 years of age in participants from that cohort who continued receiving study formulas through 1 year of age.

## Methods

Participants were originally recruited for a double-blind, controlled, parallel group, prospective, multicenter study that evaluated growth from 14 to 120 days of age as the primary objective in infants randomly assigned to receive: control EH casein formula (EHF; Nutramigen LIPIL, Mead Johnson Nutrition, Evansville, IN) or one of two investigational formulas containing LGG (10^6^ CFU/g powder; ATCC 53103, Valio, Ltd.): EH casein formula with LGG (EHF-LGG) or a PH whey/casein (60:40) formula with LGG (PHF-LGG) [35].

### Current study

Participants who completed the previous study [35] were eligible to enroll in the current study and continue with the assigned randomization number and assigned study formula through 365 days (1 year) of age. Anthropometric measures (body weight and height) were recorded, and behavioral development was assessed using selected age-appropriate milestones (adapted from [36]) through the study period (Table 1). All adverse events documented in the medical charts and those reported by the parent were recorded through 1 year of age. After 1 year of age, specific adverse events (allergy- and infection-related) and all serious adverse events were recorded.

**Table 1:** Behavioral developmental milestones from 3 to 5 years of age^a^

Milestone	3 years	5 years
Motor	● Rides tricycle	● Skips
	● Stands momentarily on one foot	
Language	● Knows age and sex	● Names four colors
	● Counts three objects correctly	● Counts ten pennies correctly
	● Repeats three numbers or sentence of six syllables	● Repeats sentence of ten syllables
Social	● Plays simple ball games (in “parallel” with other children)	● Dresses and undresses
	● Helps undressing (unbuttons clothing and puts on shoes)	● Asks questions about meanings of words
	● Washes hands	● Domestic role-playing

^a^Adapted from [2]

Parents or guardians provided written informed consent prior to enrollment. The research protocol and informed consent forms observing the Declaration of Helsinki (including October 1996 amendment) were approved by the institutional review board/ethics committee of each participating institution. The study complied with good clinical practices.

### Statistical analysis

Mean weight and height were analyzed by ANOVA. The proportion of participants in each study group who met all behavioral development milestones was compared using Fisher’s exact test. The proportion of participants in each group who experienced a specific adverse event at least one time was analyzed using Fisher’s exact test. These analyses included events occurring through 3 years of age (year 3, based on all participants with adverse event data during the second and/or third year of life) and through 5 years of age (year 5, based on all participants with adverse event data during the fourth year of life). All *P* values reported were based on two-tailed tests and considered statistically significant if <0.05. All analyses were performed using SAS version 9 (SAS Institute, Cary, North Carolina).

## Results

In the original study cohort, a total of 289 participants (EHF, 95; EHF-LGG, 95; PHF-LGG, 99) were randomized to study formula feeding groups [35]. A total of 183 participants (EHF, 63; EHF-LGG, 53; PHF-LGG, 67) were enrolled in the current study. Birth anthropometric measures (weight, length, and head circumference) and participant demographics, including smoking at home at 14 days of age and family history of allergy (data not shown), were similar among study groups. No significant differences were detected for achieved weight and height at 3 or 5 years of age (Table 2). Over 80% of infants met all of the selected milestones of behavioral development, and no group differences in milestones were detected at 3 or 5 years of age.

**Table 2:** Growth outcomes through 5 years of age^a^

Time point	Study group	Weight (kg)	Height (cm)
3 years	EHF (*n* = 37)	14.8 ± 0.3	95.5 ± 0.6
	EHF-LGG (*n* = 31)	15.5 ± 0.3	96.6 ± 0.7
	PHF-LGG (*n* = 41)	15.3 ± 0.3	96.9 ± 0.6
5 years	EHF (*n* = 28)	19.4 ± 0.5	110.5 ± 0.9
	EHF-LGG (*n* = 32)	20.8 ± 0.5	111.6 ± 0.8
	PHF-LGG (*n* = 36)	20.1 ± 0.5	111.9 ± 0.8

^a^Mean ± standard error (SE); no significant group differences by ANOVA

No significant differences in study discontinuation rates were detected with a total of 101 participants (EHF, 32; EHF-LGG, 32; PHF-LGG, 37) completing the follow-up through 5 years of age. No significant differences among groups were observed for specific allergy-related adverse events through years 3 or 5 (Table 3). The incidence of tonsillitis in the EHF-LGG compared to the PHF-LGG group and the incidence of viral skin infections in the EHF-LGG compared to the EHF group were significantly higher through year 3 (Table 4). With the exception of a significantly higher incidence of tonsillitis in the EHF-LGG compared to the PHF-LGG group, no significant differences were observed in the incidence of specific infection-related adverse events through year 5 (Table 4). No serious adverse events correlated to consumption of LGG were reported in the EH-LGG or PH-LGG group through year 5.

**Table 3:** Incidence of allergy-related adverse events through years 3 and 5^a^

	Year 3			Year 5		
Event, *n* (%)	EHF (*n* = 52)	EHF-LGG (*n* = 46)	PHF-LGG (*n* = 57)	EHF (*n* = 32)	EHF-LGG (*n* = 36)	PHF-LGG (*n* = 32)
Allergic rhinitis^b^	2 (4)	0 (0)	5 (9)	3 (9)	5 (16)	3 (8)
Allergic conjunctivitis	0 (0)	2 (4)	1 (2)	0 (0)	2 (6)	1 (3)
Allergic rhino-conjunctivitis^b^	0 (0)	0 (0)	0 (0)	1 (3)	0 (0)	0 (0)
Allergic sinusitis^b^	0 (0)	0 (0)	2 (4)	0 (0)	1 (3)	2 (6)
Wheezing	12 (23)	15 (33)	16 (28)	10 (31)	9 (28)	12 (33)
Reactive airway disease^c^	1 (2)	2 (4)	1 (2)	6 (19)	4 (13)	5 (14)
Atopic dermatitis	16 (31)	19 (41)	20 (35)	11 (34)	16 (50)	14 (39)
Contact dermatitis	2 (4)	0 (0)	4 (7)	2 (6)	1 (3)	4 (11)
Urticaria	4 (8)	3 (7)	5 (9)	5 (16)	5 (16)	3 (8)
Food Allergy	0 (0)	3 (7)	3 (5)	1 (3)	2 (6)	3 (8)
Allergic Colitis	0 (0)	1 (2)	0 (0)	0 (0)	1 (3)	0 (0)

*P* < .05; data analyzed using Fisher’s exact test

^a^No significant differences among groups

^b^Reclassified as upper respiratory infection if diagnosed in children under 2 years of age

^c^Reclassified as wheezing if diagnosed in children under 2 years of age

**Table 4:** Incidence of infection-related adverse events through years 3 and 5

	Year 3			Year 5		
Event, *n* (%)	EHF (*n* = 52)	EHF-LGG (*n* = 46)	PHF-LGG (*n* = 57)	EHF (*n* = 32)	EHF-LGG (*n* = 36)	PHF-LGG (*n* = 32)
Acute gastroenteritis	23 (44)	24 (52)	27 (47)	21 (66)	19 (59)	22 (61)
Conjunctivitis	23 (44)	21 (46)	22 (39)	19 (59)	18 (56)	16 (44)
Rhinitis	5 (10)	5 (11)	7 (12)	4 (13)	7 (22)	5 (14)
Sinusitis	13 (25)	10 (22)	16 (28)	11 (34)	16 (50)	14 (39)
Tonsillitis	1 (2)	4 (9)^a^	0 (0)	0 (0)	5 (16)^a^	0 (0)
Pharyngitis	19 (37)	16 (35)	25 (44)	17 (53)	20 (63)	23 (64)
Laryngitis	9 (17)	9 (20)	7 (12)	6 (19)	8 (25)	6 (17)
Otitis media	44 (85)	37 (80)	47 (82)	28 (88)	27 (84)	33 (92)
Upper respiratory infection	51 (98)	45 (98)	56 (98)	31 (97)	32 (100)	36 (100)
Bronchiolitis^c^	2 (4)	0 (0)	0 (0)	2 (6)	0 (0)	2 (5)
Bronchitis^d^	1 (2)	4 (9)	3 (5)	1 (3)	6 (19)	7 (19)
Pneumonia	4 (8)	4 (9)	7 (12)	2 (6)	3 (9)	7 (19)
Lower respiratory infection	6 (12)	8 (17)	10 (18)	5 (16)	8 (25)	13 (36)
Bacterial skin infection	15 (29)	10 (22)	10 (18)	13 (41)	10 (31)	11 (31)
Viral skin infection	0 (0)	4 (9)^b^	1 (2)	0 (0)	3 (9)	1 (3)
Fungal skin infection	15 (29)	13 (28)	15 (26)	10 (31)	10 (31)	12 (33)
Sepsis	2 (4)	0 (0)	0 (0)	1 (3)	0 (0)	0 (0)

Data analyzed using Fisher’s exact text

^a^Significantly different, EHF-LGG versus PHF-LGG, *P* < .05

^b^Significantly different, EHF-LGG versus EHF, *P* < .05

^c^Reclassified as wheezing if diagnosed in children under 2 years of age

^d^Reclassified as upper respiratory infection if diagnosed in children under 2 years of age

## Discussion

This study demonstrated safety for growth, development, and health events during a prolonged period of time (5 years) in children who received investigational EH and PH formulas with LGG through 1 year of age. We previously demonstrated that the investigational formulas with LGG were well tolerated and promoted adequate growth in infants from 14 through 120 days of age [35]. In the current study, both investigational formulas with LGG were associated with normal growth and development through 5 years of age, as well as absence of relevant infections or allergic events, or serious adverse events that could be attributed to early consumption of LGG.

CMA and associated allergic manifestations may increase energy requirements through skin, gastrointestinal or respiratory inflammation, disrupted sleep patterns, and reduced absorption of major nutrients [37]. Assessment of suitability of EH formula for growth in infants and children with CMA can be challenging, given the impact of the allergic condition on growth regardless of nutrient source. For example, significantly lower mean weight-for-height has been reported in children with atopic eczema compared to children without atopic eczema in a follow-up study through 4 years of age [14]. In healthy children with a family history of allergy who received EH casein formula through 4 months of age, slower BMI gain was reported in the first year of life but no differences in BMI were detected by 6 years of age [38]. Likewise, results from the current study indicated that all participants who received EH or PH formulas exhibited normal growth (weight and height) by 5 years of age.

Infant formula with LGG has been available for many years. This is the first study evaluating long-term growth and safety of children fed formula with LGG through 1 year of age. Perinatal administration of LGG through 6 months of age to infants with a family history of allergy has been associated with no health concerns and normal growth (height and weight-for-height) [28]; it also restrained overweight, as indicated by reduced birth weight-adjusted BMI at 4 years of age [32]. A routine cow’s milk-based formula with LGG promoted normal growth in healthy-term infants through 6 months of age compared to formula without LGG [55]. In healthy-term infants, we previously demonstrated that an EH casein formula with LGG transiently colonized the intestinal tract and was well tolerated and associated with normal growth through 120 days of age and with circulating fatty acids similar to those of breastfed infants [40] [47]. Results of the current study extend safety for growth and tolerance of EH and PH formulas with LGG through 5 years of age in a healthy population and lead to the conclusion that early LGG supplementation is associated not only with normal infant growth but also with healthy weight gain later in life.

The probiotic LGG has nearly 30 years of safe use. Risk associated with LGG is considered low, though questions associated with live probiotic bacteria use, particularly in immunocompromised and critically ill individuals, still arise [34, 39, 40]. LGG has demonstrated good safety profile in those individuals, with no report of septicemia or other serious adverse events [41–43]. In elderly people with declining immune competence, dietary LGG was demonstrated to be safe and well tolerated [44] and retrospective analyses of routine LGG use in premature infants for a 6-year period in Italy [42] and a 12-year period in Finland [43] demonstrated safety of LGG. An isolated study reported an increased incidence of wheezing in a group of high-risk infants who received perinatal supplementation with LGG compared to placebo, based on physician diagnosis or parental report up to 2 years of age [26]. In the current study, allergic and infectious adverse events were compared among groups for a longer follow-up period and no differences in wheezing were observed. We did not observe an increase in any allergy-related events in the groups receiving formulas supplemented with LGG. In fact, one of the potential benefits of probiotic use is increased tolerance to various allergens via modulation of the gut immune system and reinforcement of the intestinal mucosal barrier. Infants with CMA developed an accelerated acquisition of tolerance to cow’s milk after receiving EH formula with LGG (compared to an EH formula without LGG) over a 12-month period [30, 45]. LGG supplementation has also demonstrated to decrease incidence of AD in high-risk population [29]. In the present study, the incidence of AD through years 3 and 5 was not significantly different among study groups. However, our population was not selectively comprised of infants at high risk of allergy and had a smaller sample size, both of which might explain the different outcomes.

Protection against gastrointestinal and respiratory infections is among the potential benefits of probiotics and notably LGG [25, 46–48]. Such protection was not detected in our study, nor was a deleterious effect of LGG. The incidence of tonsillitis through years 3 and 5 was higher in the EHF-LGG but not the PHF-LGG group. Likewise, the incidence of viral skin infections was higher in the EHF-LGG but not the PHF-LGG group through year 3, with no significant differences through year 5. Therefore, the differences were not consistently associated with the LGG-enriched formulas and thus unlikely related to the presence of LGG in the investigational formulas. In addition, the incidence of tonsillitis in the EHF-LGG is within the incidence of tonsillitis normally reported in this age group [49, 50]. Likewise, the incidence of viral skin infections reported in the EHF-LGG group through year 3 is within the expected incidence of these conditions in young children [51, 52]. Consequently, in the current healthy study population, the few significant differences observed in the incidence of specific infection-related adverse events are not considered clinically relevant.

Age-appropriate behavioral development is a valuable indicator of long-term health and safety of early use of LGG. Behavioral development may be influenced by multiple biological and environmental factors; however, age-appropriate milestones assessing motor, language, and social skills can appropriately evaluate development [36]. In the present study, the great majority of infants met all developmental milestones and no group differences were detected at 3 and 5 years of age, demonstrating that study formulas supported normal behavioral development through 5 years of age. Recent evidence suggests that gut microbiota may impact neurocognitive outcomes via the gut-brain-axis [47]. A study with preterm infants demonstrated that early probiotic supplementation significantly reduced infant crying and fussiness, indicating that probiotic supplementation may reduce excessive crying and irritability in infants [53]. A study in colicky term infants also suggests that a combination of behavioral counseling, cow’s milk elimination diet, and LGG supplementation may decrease the number of crying days [54]. Furthermore, a reduced prevalence of attention-deficit hyperactivity disorder and Asperger syndrome at 13 years of age was observed in children who received LGG through 6 months of age [55].

The use of LGG in EH formulas is a promising option due to the potential of faster acquisition of tolerance to cow’s milk protein in infants with CMA. Results of this study indicate that extensively and partially hydrolyzed formulas with LGG, when consumed by healthy-term infants through 1 year of age, are associated with normal growth and development and long-term safety through 5 years of age.

## Abbreviations

AD: Atopic dermatitis
ANOVA: Analysis of variance
CMA: Cow’s milk allergy
CMP: Cow’s milk protein
EH: Extensively hydrolyzed
LGG: *Lactobacillus rhamnosus*
PH: Partially hydrolyzed
